# Variable retinal presentations after retrobulbar needle perforation: a case series

**DOI:** 10.1186/s40942-026-00897-0

**Published:** 2026-07-30

**Authors:** Eva Ramón Juan, David Oliver-Gutiérrez, Alberto Salmoral Lorenzo-Arroyo, Claudia Garcia-Arumí, José Garcia-Arumí

**Affiliations:** 1 General Ophthalmology Department, IMO Grupo Miranza, Balmes, 322, Barcelona, 3 1. 08006 Spain; 2 Retina and Vitreoretinal Surgery Department, Hospital Universitari Bellvitge (HUB), Barcelona, Spain; 3 Retina and vitreoretinal Surgery Department, IMO Grupo Miranza, Barcelona, Spain; 4 Retina and vitreoretinal Surgery Department, Hospital Universitari Vall d’Hebrón (HUVH), Barcelona, Spain

**Keywords:** Retrobulbar anesthesia, Globe perforation, Retinal injury, Needle injury, Retinal perforation, Submacular hemorrhage, Choroidal neovascularization, Proliferative vitreoretinopathy, Iatrogenic ocular trauma

## Abstract

**Background:**

Retrobulbar anesthesia is widely used in ophthalmic surgery due to its rapid onset and effective akinesia. However, although uncommon, globe perforation remains one of its most severe complications and may lead to a wide spectrum of posterior segment damage. While incidence and risk factors have been extensively described, less attention has been given to the management of the resulting retinal and choroidal injuries.

**Main body:**

We report a retrospective case series of four patients with retinal or choroidal complications following retrobulbar needle perforation. Clinical presentations were heterogeneous, including proliferative vitreoretinopathy, subretinal hemorrhage, choroidal neovascularization, and submacular hemorrhage. Management was individualized according to the predominant retinal pathology and included pars plana vitrectomy–based approaches, subretinal recombinant tissue plasminogen activator, and intravitreal anti-vascular endothelial growth factor therapy. Anatomical stabilization was achieved in all cases, with visual outcomes appearing to be influenced by macular involvement and timing of intervention.

**Conclusion:**

Retrobulbar needle perforation can result in diverse retinal pathologies despite a common mechanism of injury. This small case series illustrates the importance of early recognition, multimodal imaging, and individualized management in selected complex cases.

## Background

Retrobulbar anesthesia (RBA) remains one of the most widely used regional anesthetic techniques in ophthalmic surgery due to its rapid onset, effective akinesia, and reliable analgesia. Despite its overall safety profile, it is not exempt from serious complications [1, 2].

Although peribulbar anesthesia was introduced to avoid the risks associated with retrobulbar anesthesia, the complication rates of the two techniques are similar [3–5]. Among the most severe adverse events are globe perforation and retrobulbar hemorrhage, both rare but potentially vision-threatening. Large series report an incidence of globe perforation ranging from approximately 0.006% to 0.13% [4], while retrobulbar hemorrhage has been described in 0.04% to 0.44% of cases [2, 6]. These complications may lead to irreversible visual loss if not promptly recognized and appropriately managed.

While numerous publications have addressed incidence, risk factors, and preventive strategies, comparatively little attention has been devoted to the management of posterior segment complications once perforation has occurred [7, 8]. Needle-related ocular injury may result in a broad spectrum of retinal and choroidal manifestations, including vitreous hemorrhage, retinal tears, subretinal hemorrhage, retinal detachment and macular involvement, which may require individualized surgical decision making.

In this report, we present four representative cases illustrating how a single iatrogenic mechanism can produce distinct anatomical patterns: the resulting pathology ranged from acute submacular hemorrhage to secondary choroidal neovascularization (CNV), localized proliferative vitreoretinopathy (PVR), and recurrent retinal detachment. This heterogeneity highlights the importance of individualized management. This study aims to characterize different retinal injury patterns following retrobulbar needle perforation and to illustrate their corresponding management approaches.

## Main text

## Materials and methods

We conducted a retrospective review of four consecutive patients who developed retinal or choroidal injury following retrobulbar anesthesia and were managed at our center between 2013 and 2025. Clinical data, imaging findings, surgical management, and outcomes were reviewed.

Cases were identified through retrospective review of surgical records and referred patients presenting to our center with retinal or choroidal complications following recent ocular surgery under retrobulbar anesthesia. Attribution to retrobulbar needle perforation was based on a combination of clinical history, temporal relationship with anesthesia, and characteristic intraoperative or imaging findings, including focal scleral lesions, subretinal hemorrhage patterns, and absence of alternative causes.

The site of perforation was determined based on clinical examination, multimodal imaging, and intraoperative findings, and was considered presumed when not directly visualized.

Information regarding the operator performing the retrobulbar anesthesia was available only for the case managed at our institution; in referred cases, detailed data on operator experience (junior vs. senior) were not available due to the retrospective nature of the study. The study adhered to the tenets of the Declaration of Helsinki and was conducted following institutional approval.

## Results (case series)

Four patients were included. Clinical presentations and management strategies are summarized in Table 1.

### Case 1

A 64-year-old male presented two months after pars plana vitrectomy for rhegmatogenous retinal detachment in the left eye (LE), reporting persistent metamorphopsia and a central scotoma since surgery. Best-corrected visual acuity (BCVA) was 0.5 logMAR.

**Fig. 1:**
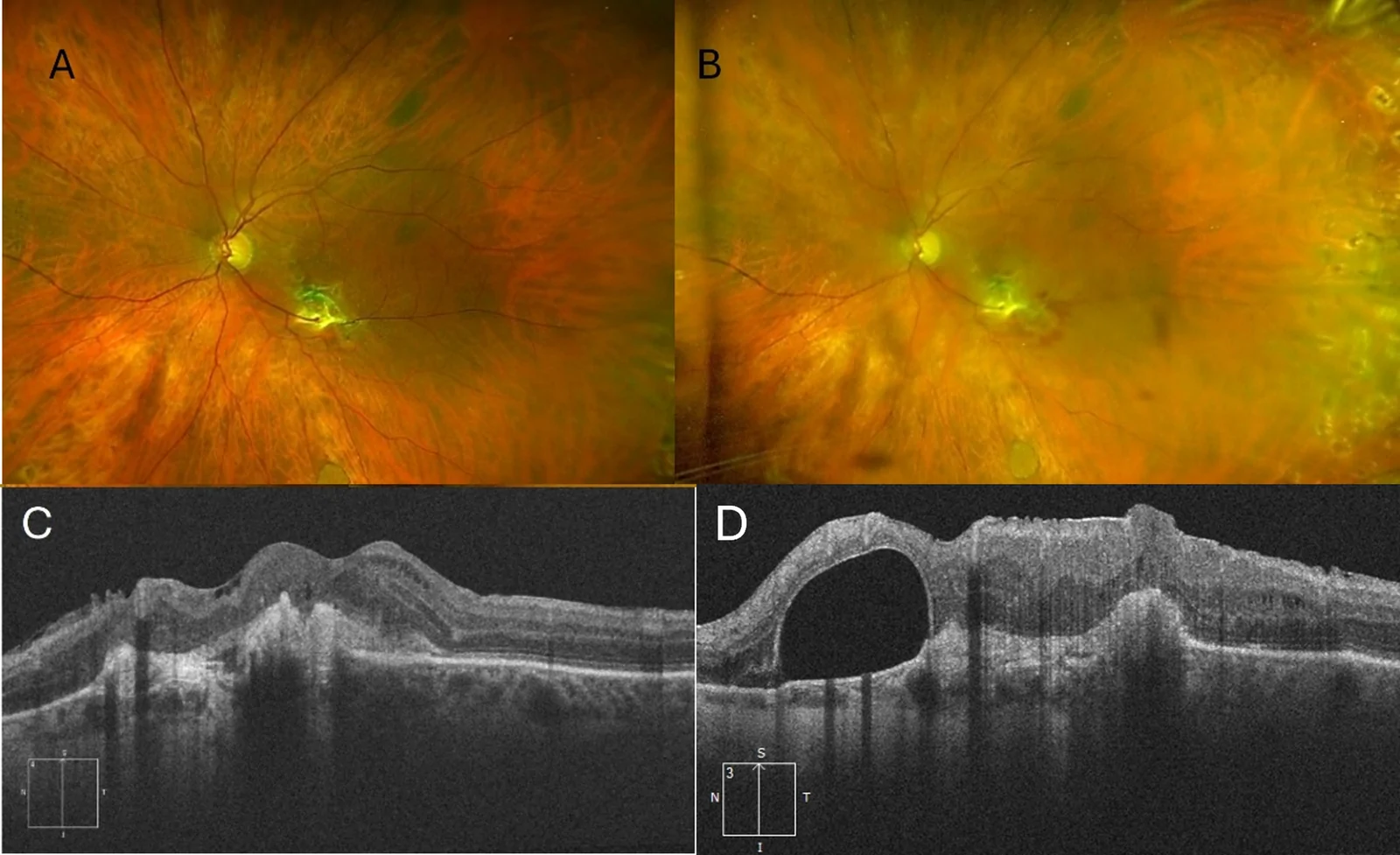
Preoperative findings (Case 1).** A–B**. Ultra-widefield fundus imaging showing localized macular PVR along the inferotemporal arcade. **C-D**. OCT demonstrating epiretinal traction with macular distortion and subretinal hyperreflective material consistent with retained PFCL and secondary neovascularization

Widefield fundus imaging revealed localized macular proliferative vitreoretinopathy (PVR) surrounding the inferior temporal arcade, associated with residual subretinal blood and retained subretinal perfluorocarbon liquid (PFCL) (Fig. 1A–D). Spectral-domain optical coherence tomography (OCT) demonstrated a tractional epiretinal membrane with macular distortion and hyperreflective subretinal material at the presumed needle entry site, consistent with secondary choroidal neovascularization (Fig. 1C).

**Fig. 2:**
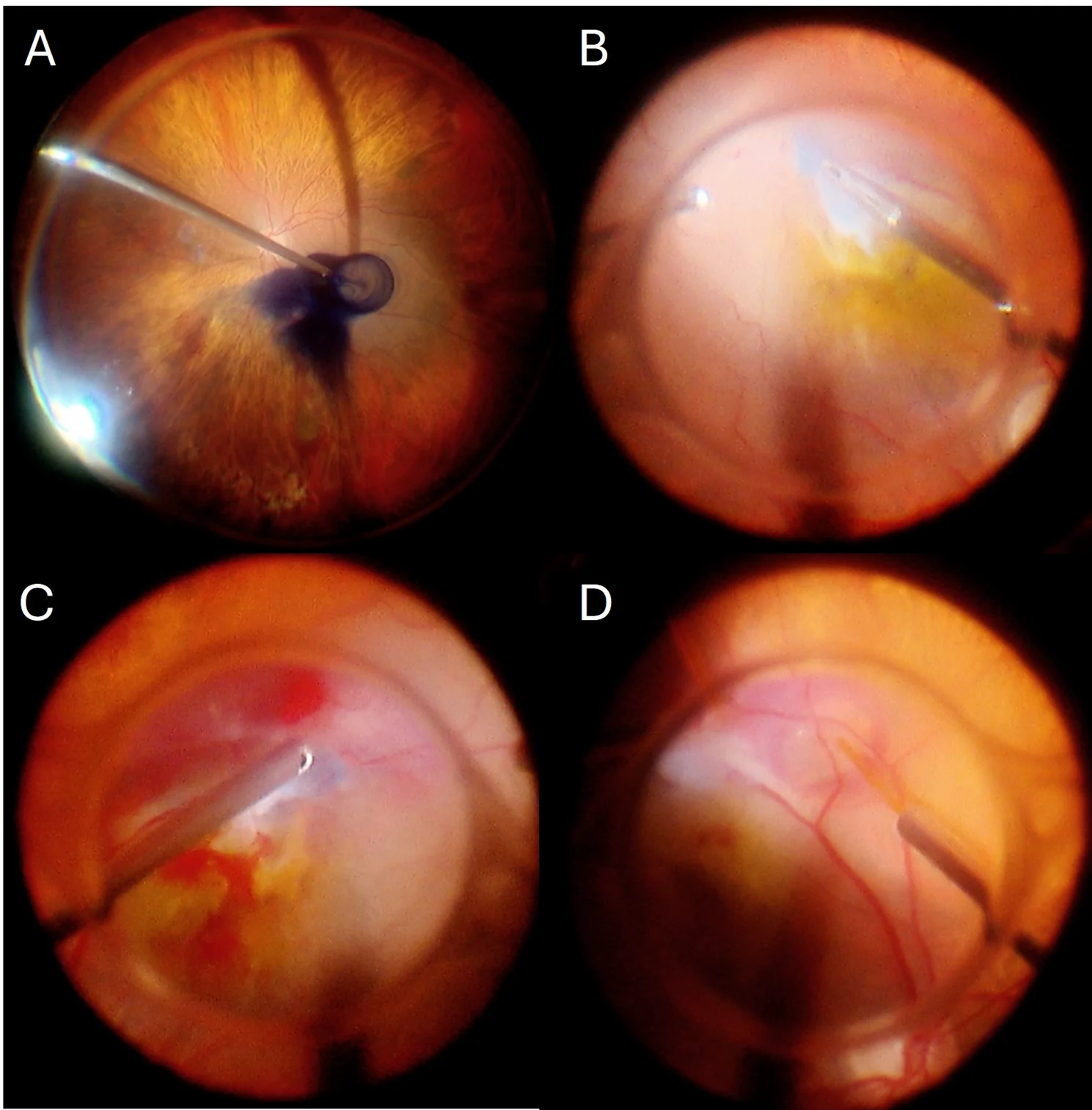
Intraoperative management of macular PVR (Case 1).** A**. Macular staining with Dual Blue^®^. **B**. Bimanual dissection of epiretinal proliferation. **C**. Drainage of subretinal fluid through a retinal defect. **D**. Removal of subretinal perfluorocarbon liquid using a 41-gauge cannula

Given the macular traction and presence of subretinal material, repeat pars plana vitrectomy was performed, including membrane peeling and removal of retained PFCL (Fig. 2A-D). At six months, BCVA was 0.5 logMAR. Fundus examination showed a well-demarcated chorioretinal scar along the inferior temporal artery corresponding to the presumed needle tract, with established submacular fibrosis.

### Case 2

A 43-year-old male presented with hand-motion vision in the right eye (RE) due to a total retinal detachment. Combined scleral buckling and pars plana vitrectomy were planned. During induction of retrobulbar anesthesia, globe perforation occurred, resulting in acute hypotony.

**Fig. 3:**
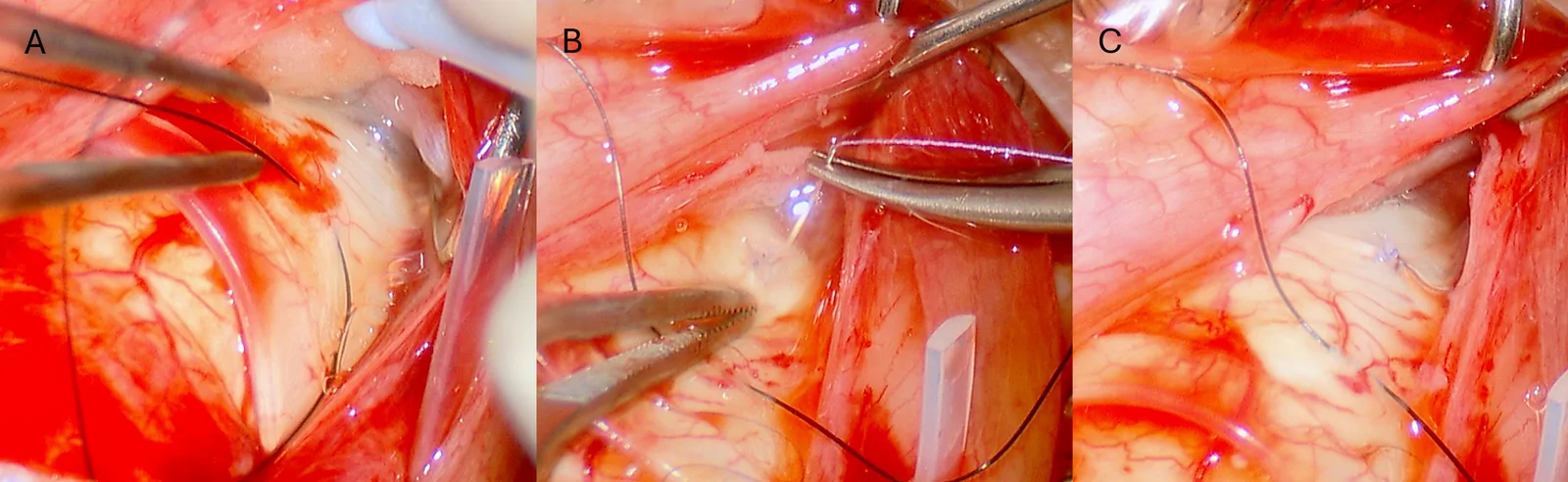
Scleral repair of needle perforation (Case 2).** A**. Intraoperative identification of presumed needle entry site. **B**. Closure with absorbable sutures under hypotonic conditions

Intraocular pressure was restored with balanced salt solution (BSS) injected into the anterior chamber and vitreous cavity. The presumed scleral entry site was identified based on focal scleral hemorrhage at a posterior location, clearly posterior to the intended buckle placement, and was closed with absorbable sutures. (Fig. 3).

**Fig. 4:**
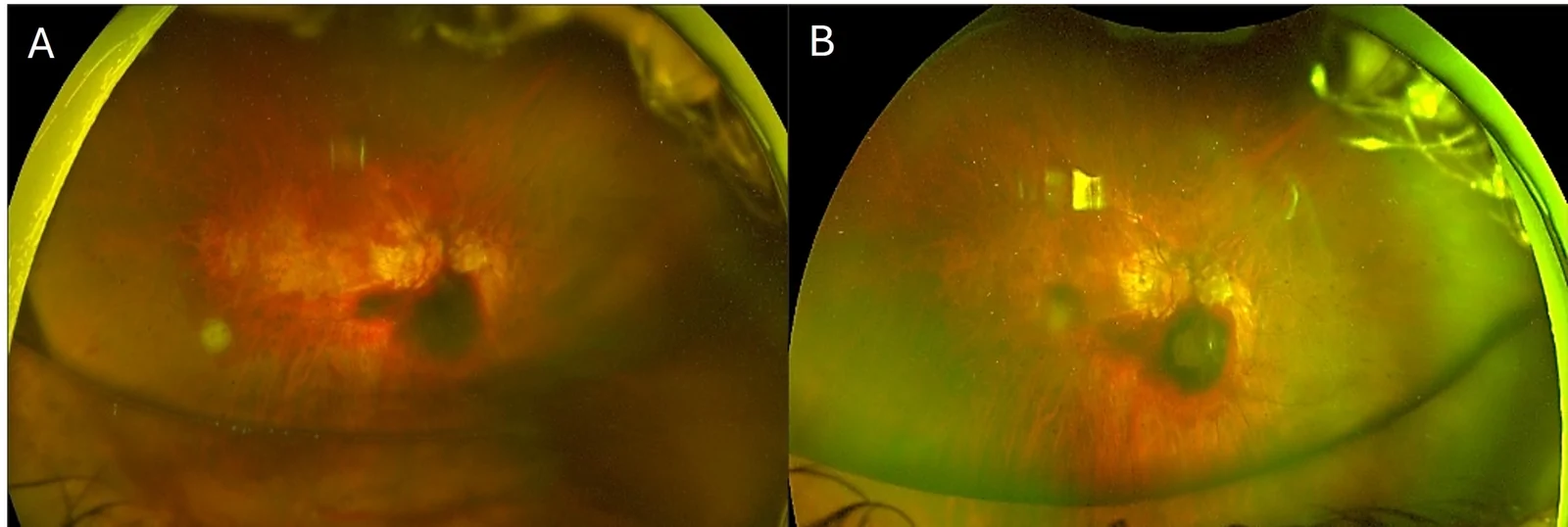
Intraoperative retinal stabilization (Case 2).** A**. PFCL used to stabilize the posterior pole. **B**. Final appearance after retinotomy, subretinal fluid drainage, and silicone oil tamponade

Intraoperatively, a localized subretinal hemorrhage was observed inferior to the macular area (Fig. 4). Because of the proximity of the hemorrhage to the macula, the initial surgical plan was modified. Scleral buckling was deferred and the procedure was rapidly converted to an intraocular approach in order to stabilize the posterior pole and prevent posterior extension of the hemorrhage.

Perfluorocarbon liquid (PFCL) was used to flatten the detached retina and limit posterior displacement of the subretinal blood. A superior retinotomy at 12 o’clock allowed controlled drainage of subretinal fluid, followed by fluid–air exchange and silicone oil tamponade (5000 cSt).

During the first postoperative month, silicone oil emulsification with elevated intraocular pressure required a second surgery consisting of silicone oil removal combined with phacoemulsification and anterior chamber washout.

Three weeks later, the patient developed recurrent ocular hypertension and dense vitreous hemorrhage. B-scan ultrasonography suggested inferior retinal redetachment, and a third surgery revealed multiple tractional retinal tears consistent with proliferative vitreoretinopathy secondary to the initial perforation and intraocular hemorrhage.

At final follow-up two years later, BCVA improved to 1.0 logMAR, with a stable attached retina.

### Case 3

A 72-year-old woman presented with BCVA of 1.3 logMAR in the RE. Her ocular history included vitrectomy with gas tamponade 4 months prior for a macular hole in the RE.

**Fig. 5:**
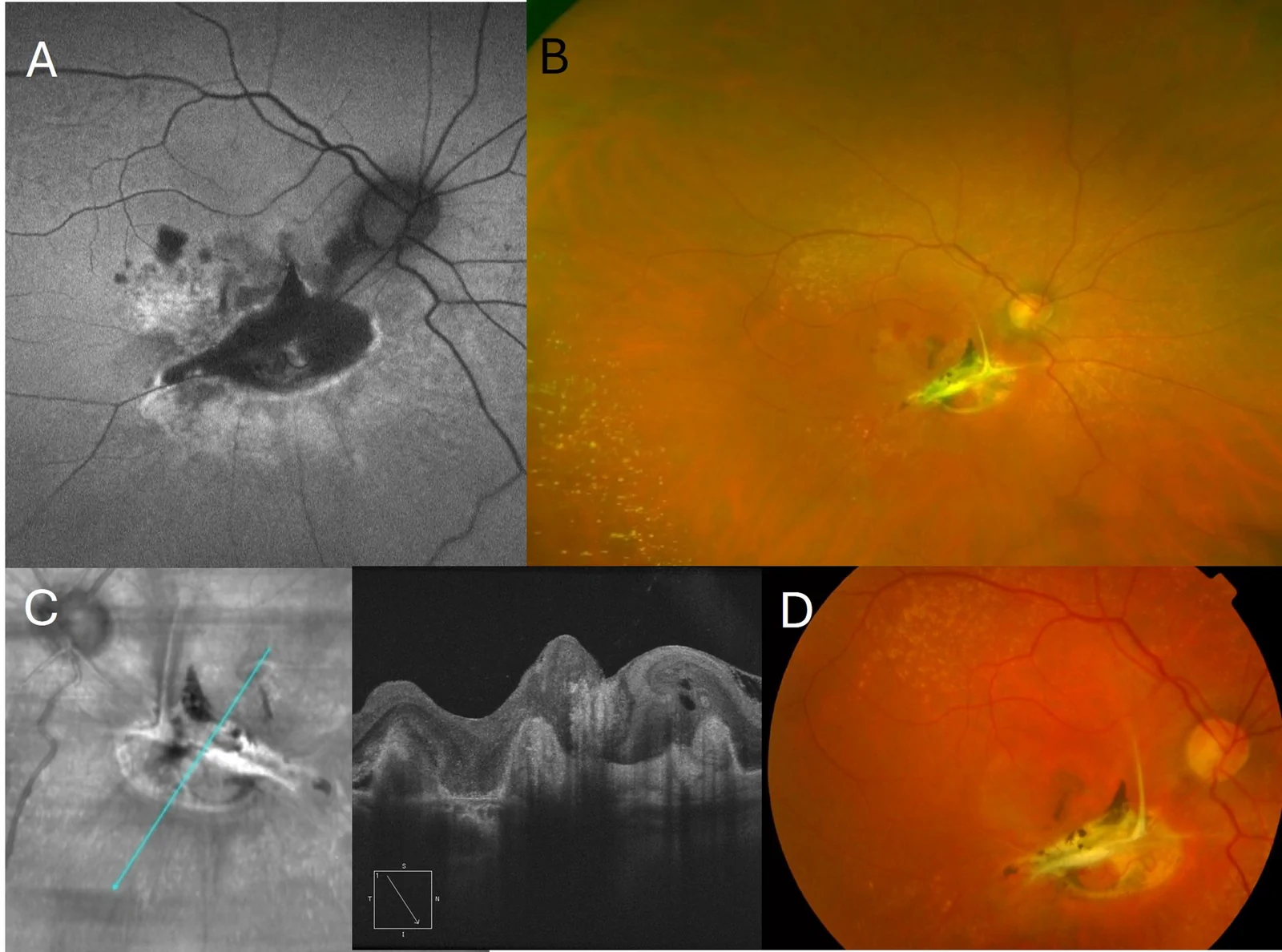
Postoperative findings with CNV (Case 3).** A–B**. Fundus photography and autofluorescence showing subfoveal fibrosis and RPE alterations. **C**. OCT demonstrating outer retinal disruption with subretinal hyperreflective material. **D**. Residual fibrotic scar after anti-VEGF therapy

She subsequently developed a macular hemorrhage associated with choroidal neovascularization at the presumed needle injury site, along with subfoveal fibrosis and tractional changes (Fig. 5A-B). OCT confirmed active CNV (Fig. 5C).

She was treated with five intravitreal injections of bevacizumab over four months, resulting in anatomical stabilization (Fig. 5D) and improvement of BCVA to 0.7 logMAR.

### Case 4

A 60-year-old woman with a history of bilateral retinal detachment repair underwent pars plana vitrectomy for epiretinal membrane in the LE. The procedure had been performed under retrobulbar anesthesia. Six days postoperatively, she presented with counting fingers at 25 cm and an inferior visual field defect.

**Fig. 6:**
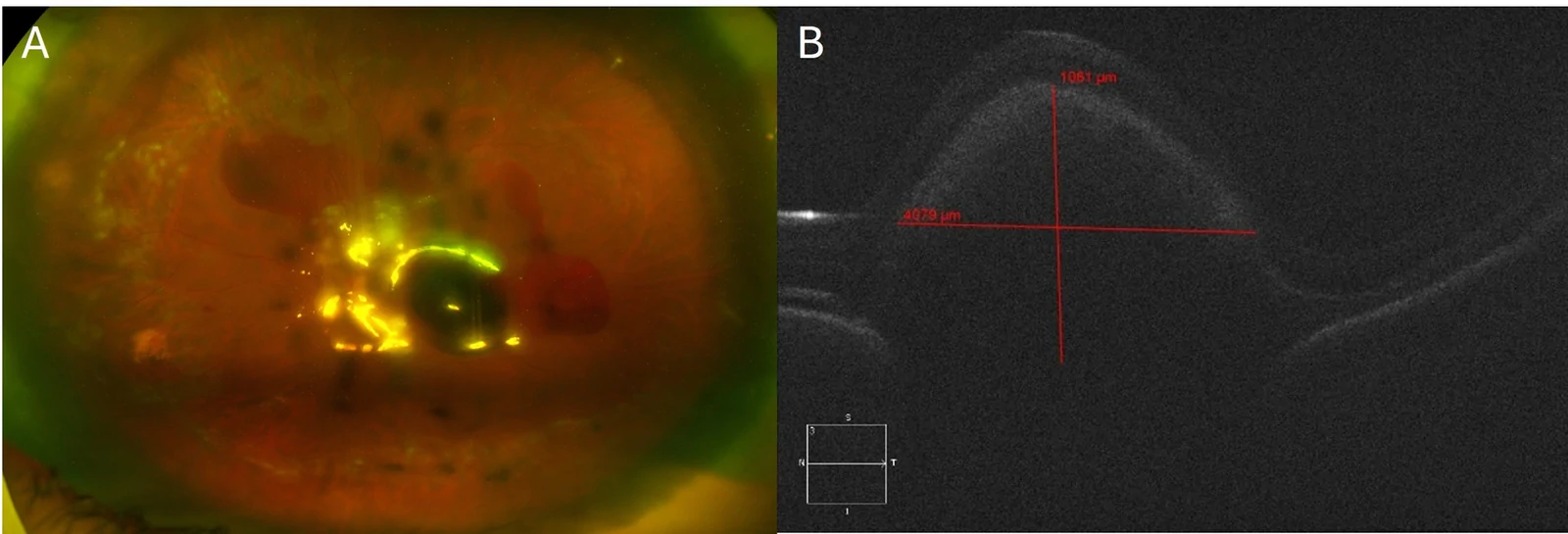
Submacular hemorrhage (Case 4).** (A)** Dense submacular hemorrhage. **(B)** OCT showing thick subfoveal clot (~ 1000 μm)

Fundus examination revealed a dense submacular hemorrhage involving the entire macula, with additional patchy subretinal hemorrhages in the mid-periphery (Fig. 6A). Spectral-domain OCT demonstrated a thick subfoveal clot measuring approximately 1000 μm in vertical height.

**Fig. 7:**
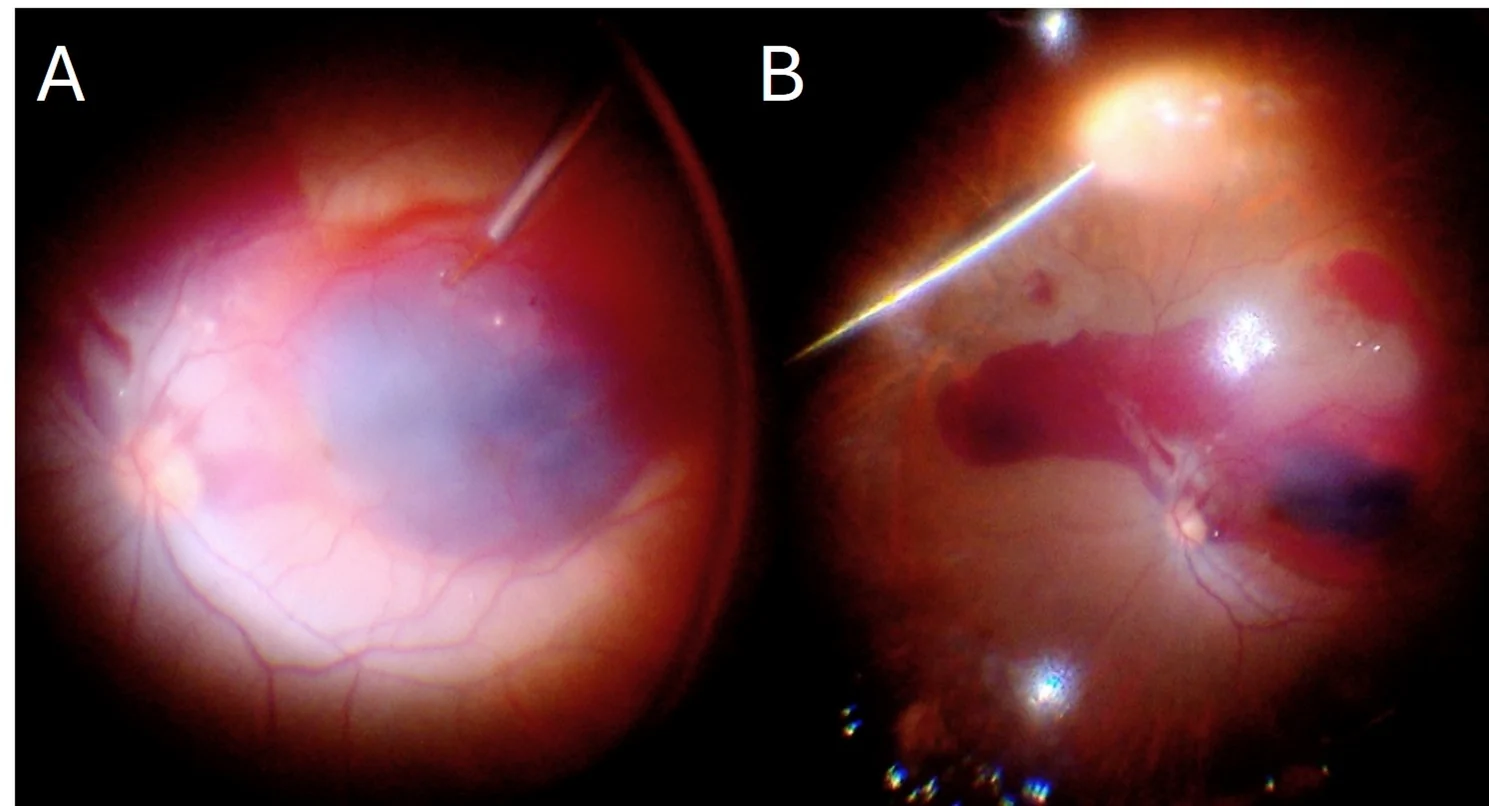
Subretinal rTPA injection (Case 4).** A**. Subretinal injection of rTPA inducing a localized retinal detachment. **B**. Early displacement of submacular hemorrhage

Given the substantial thickness of the hemorrhage (Fig. 6B), surgical displacement was indicated. An urgent 25-gauge pars plana vitrectomy was performed. A small peripheral retinotomy was created in an area of subretinal hemorrhage away from the fovea. Using a 41-gauge flexible cannula, recombinant tissue plasminogen activator (rTPA) was delivered into the subretinal space with the aim of creating a cleavage plane between the retinal pigment epithelium and the clot to facilitate displacement (Fig. 7). After adequate diffusion, complete fluid–air exchange was performed, followed by 20% sulfur hexafluoride (SF₆) gas tamponade. Postoperatively, the patient remained seated for 45 min to allow gravitational displacement of liquefied blood, followed by prone positioning to promote inferior displacement.

**Fig. 8:**
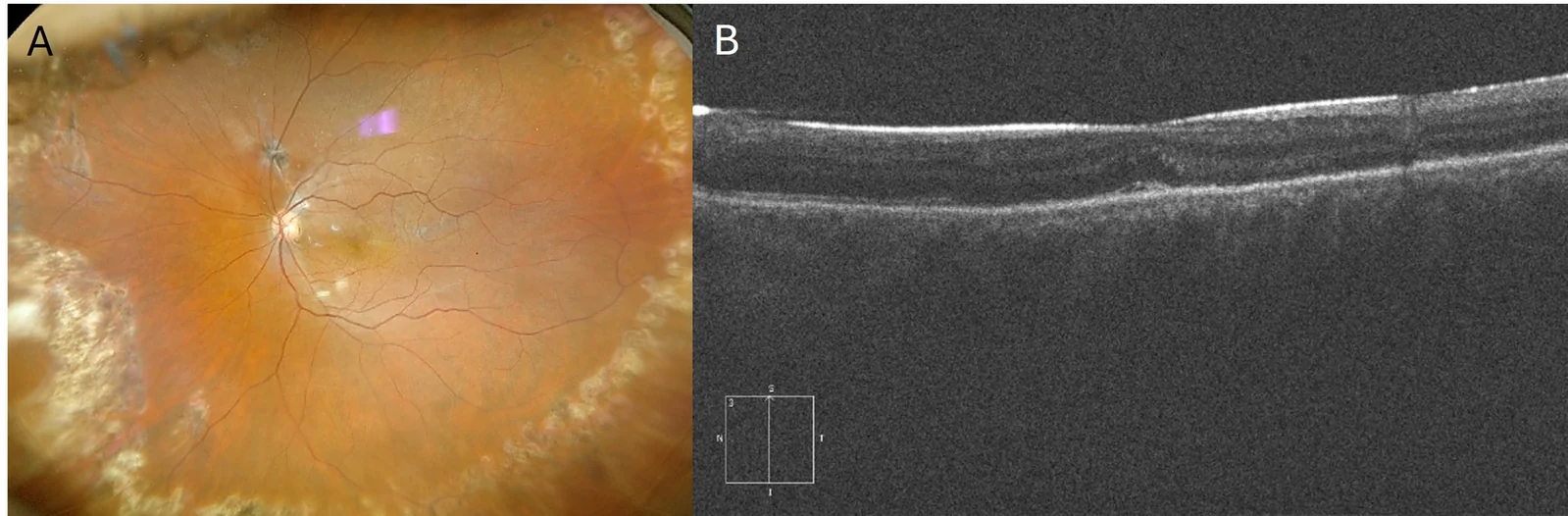
Postoperative recovery (Case 4).** (A)** Fundus image after hemorrhage displacement. **(B)** OCT showing restoration of normal foveal contour

Three weeks later, BCVA improved to 0.4 logMAR, with significant foveal clearing on fundus examination (Fig. 8A). Six months thereafter, cystoid macular edema secondary to progressive epiretinal membrane formation developed, requiring repeat vitrectomy with epiretinal and internal limiting membrane peeling under Dual Blue^®^ (0.15% trypan blue + 0.025% brilliant blue G; DORC International, Zuidland, The Netherlands). Four months after the final procedure, BCVA improved to 0.05 logMAR, with restored macular contour and stable OCT findings (Fig. 8B).

**Table 1:** Site of perforation refers to the presumed location based on clinical and imaging correlation

Case	Age/Sex	Initial BCVA (logMAR)	Time of Diagnosis	Site of perforation	Axial length (mm)	Main complication	Surgical approach	Final BCVA (logMAR)	Follow-up duration (months)
1	64/Male	0.52	Postoperative (2 months)	Presumed inferotemporal posterior pole (along inferior temporal arcade)	22.5	Macular PVR with retained PFCL and CNV	PPV + membrane peeling	0.52	6
2	43/Male	2.3	Intraoperative (recognized immediately)	Presumed inferoposterior pole	25	Subretinal hemorrhage after perforation	PPV + PFCL + silicone oil	1.0	24
3	72/Female	1.3	Postoperative (4 months)	Presumed subfoveal / juxtafoveal	Not available	CNV with subfoveal fibrosis	Not surgical (Anti-VEGF)	0.7	4
4	60/Female	1.8	Postoperative (6 days)	Presumed posterior pole (submacular involvement)	Not available	Thick submacular hemorrhage	PPV + subretinal rTPA	0.05	10

## Discussion

Needle perforation represents a trans-scleral, multi-layer injury triggering hemorrhagic, inflammatory, fibrotic, and angiogenic cascades [9–11]. Subretinal blood exposure, vitreous inflammation, and mechanical tissue disruption contribute to fibrocellular proliferation and traction [12], while focal breaks in Bruch’s membrane and choriocapillaris damage predispose to CNV formation. These mechanisms may contribute to the spectrum of retinal phenotypes observed in our series.

Temporal factors play a critical role in determining both the clinical presentation and the management strategy. When perforation is recognized intraoperatively, immediate restoration of intraocular pressure, closure of the scleral entry site, and retinal stabilization with perfluorocarbon liquid are essential to prevent posterior extension of subretinal hemorrhage, allowing prompt adjustment of the surgical plan (Case 2). In contrast, delayed presentations are typically driven by secondary processes such as fibrosis, membrane formation, or angiogenic activation. Early submacular hemorrhage may benefit from prompt subretinal rTPA administration combined with pneumatic displacement, aiming to reduce photoreceptor toxicity [13, 14]. Delayed diagnosis may allow subretinal blood to involve the posterior pole, requiring active displacement with subretinal rTPA. Chronic cases often require complex vitreoretinal surgery or anti-vascular endothelial growth factor therapy, depending on the predominant phenotype. Timing of diagnosis appears to influence both the anatomical pattern and the subsequent management approach.

While previous reports have primarily focused on incidence and risk factors associated with retrobulbar anesthesia–related perforation [4, 15], detailed descriptions of management strategies tailored to specific anatomical and biological patterns remain limited. In our limited series, visual outcomes appeared to be influenced by the degree and duration of macular involvement; however, given the small sample size and heterogeneity of presentations, no definitive clinicopathologic correlations can be established.

A relevant clinical consideration is that a significant proportion of cases may remain unrecognized at the time of the anesthetic injection. In the referred cases in our series (all except case 2), the type of needle used for retrobulbar anesthesia was unknown. Previous studies have demonstrated that globe perforation may occur with both sharp and blunt needles [16]. Retrobulbar anesthesia has been suggested to be avoided in patients with high axial myopia, due to the increased risk of scleral perforation [15]. Interestingly, contrary to what might be intuitively expected, the most frequently reported site of perforation is posterior, often in proximity to the macular region or even beneath the fovea [17].

Previous studies emphasize that early intraoperative recognition of globe perforation is crucial to prevent severe complications such as retinal detachment or vitreous hemorrhage [8]. The presence of unexplained vitreous hemorrhage during postoperative evaluation should prompt careful investigation [18]. When the media are clear and a retinal break is identified, laser photocoagulation around the perforation site may be sufficient if no additional complications are present. Importantly, long-term follow-up remains essential, as delayed accumulation of subretinal fluid has been reported [8].

Cases with multiple perforations, posterior breaks, or those associated with retinal detachment and proliferative vitreoretinopathy require complex vitreoretinal surgery and are associated with a poorer visual prognosis [18]. The presence of vitreous hemorrhage, with or without retinal detachment, often necessitates early pars plana vitrectomy, as proliferative vitreoretinopathy may develop rapidly and aggressively.

This study has several limitations. Its retrospective design may have led to underrepresentation of milder cases, and the inclusion of only patients referred to a tertiary care center introduces a potential bias toward more severe presentations. Attribution of retinal and choroidal findings to retrobulbar needle perforation was based on clinical and intraoperative correlation and could not always be directly confirmed. In addition, the limited sample size and heterogeneity of findings preclude the definition of a standardized management algorithm. However, in contrast to many published series focused mainly on incidence and risk factors, this study emphasizes detailed clinical characterization and the management approach. Therefore, the findings should be interpreted as illustrative of possible clinical patterns rather than definitive management recommendations.

## Conclusions

Globe perforation during retrobulbar anesthesia, although uncommon, can result in a wide spectrum of retinal and choroidal pathology. These cases highlight that early recognition, thorough multimodal imaging, and individualized management based on anatomical and temporal factors are important to optimize visual outcomes. These findings suggest that retrobulbar needle perforation may be considered a dynamic retinal injury in which clinical presentation varies according to anatomical and temporal factors, requiring individualized management approaches.

## Data Availability

Not applicable.

## Abbreviations

LE: Left eye
RE: Right eye
BCVA: Best-corrected visual acuity
CNV: Choroidal neovascularization
OCT: Optical coherence tomography
PFCL: Perfluorocarbon liquid
PPV: Pars plana vitrectomy
PVR: Proliferative vitreoretinopathy
RBA: Retrobulbar anesthesia
rTPA: Recombinant tissue plasminogen activator
SF₆: Sulfur hexafluoride
BSS: Balanced salt solution
RPE: Retinal pigmented epithelium
